# Emerging therapeutic targets in esophageal adenocarcinoma

**DOI:** 10.18632/oncotarget.8777

**Published:** 2016-04-17

**Authors:** Puja Gaur, Clayton R. Hunt, Tej K. Pandita

**Affiliations:** ^1^ Department of General Surgery, Division of Thoracic Surgery, The Houston Methodist Research Institute, Houston, TX, USA; ^2^ Department of Radiation Oncology, The Houston Methodist Research Institute, Houston, TX, USA

**Keywords:** esophageal adenocarcinoma, cancer stem cells, immunotherapy, genetic and epigenetic targets, chemoradioresistance

## Abstract

The incidence of gastro-esophageal disease and associated rate of esophageal adenocarcinoma (EAC) is rising at an exponential rate in the United States. However, research targeting EAC is lagging behind, and much research is needed in the field to identify ways to diagnose EAC early as well as to improve the rate of pathologic complete response (pCR) to systemic therapies. Esophagectomy with subsequent reconstruction is known to be a morbid procedure that significantly impacts a patient's quality of life. If indeed the pCR rate of patients can be improved and those patients destined to be pCR can be identified ahead of time, they may be able to avoid this life-altering procedure. While cancer-specific biological pathways have been thoroughly investigated in other solid malignancies, much remains unexplored in EAC. In this review, we will highlight some of the latest research in the field in regards with EAC, along with new therapeutic targets that are currently being explored. After reviewing conventional treatment and current changes in medical therapy for EAC, we will focus on unchartered grounds such as cancer stem cells, genetics and epigenetics, immunotherapy, and chemoradio-resistant pathways as we simultaneously propose some investigational possibilities that could be applicable to EAC.

## INTRODUCTION

**Figure 1:**
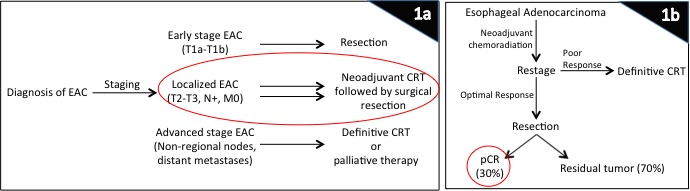
Treatment of esophageal cancer solely depends on staging For early-stage tumors without any nodal involvement, patients are referred directly to surgical resection, while patients with advanced tumors and distant metastases are committed to definitive chemoradiation or palliation. However, those patients who have localized disease without any distant metastases, standard of care includes neoadjuvant chemoradiation therapy (1a). Those patients who have a favorable response to chemoradiation on restaging then undergo a surgical resection, and about 30% of these patients have a pathological complete response (1b). This group of patients have the best long-term outcome in terms of recurrence, metastases, and overall as well as disease-free survival. Abbreviations: CRT, chemoradiation therapy; EAC, esophageal adenocarcinoma; pCR, pathologic complete response.

Esophageal cancer remains the sixth most commonly occurring form of cancer and continues to be an aggressive cancer due to its diagnosis in late stages. Overall 5-year survival for patients with esophageal cancer remains dismal at 18% according to the latest statistics, despite clinical advances [1, 2]. In 2015 alone, there will be a predicted 16,980 patients diagnosed with esophageal cancer in the United States with nearly equal number of deaths [2]. Unfortunately, due to lack of a universal screening tool and the advanced stage at which symptoms develop [3], only about half of the patients are considered resectable at the time of diagnosis. While patients with early stage tumors (T1a-T1b) are appropriate candidates for resection, locally advanced tumors (T2-T3, node positive, of either histologic subtype) are treated with upfront chemoradiation followed by surgical resection after restaging as per the CROSS group recommendations (chemoradiotherapy for oesophageal cancer followed by surgery study, Figure 1a) [4]. This trimodality therapy where patients are treated with neoadjuvant chemotherapy and radiation followed by surgery offers the best clinical outcome with a 26-33% complete response rate for these locally advanced tumors (Figure 1b) [5, 6]. Indeed, those patients with pathologic complete response (pCR) have much better prognosis in terms of recurrence, metastatic potential, and long-term survival [7]. With the rising incidence of gastroesophageal reflux and an epidemic in Barrett's esophagus and esophageal adenocarcinoma (EAC) in the United States, it is imperative that we understand the molecular biology of this disease in order to strategically develop biological therapies and to simultaneously develop an effective pre-symptomatic screening tool. In this review, we will discuss current and new therapeutic targets for EAC and suggest some potential preclinical models to provide a resource for identification of biomarkers that can be exploited for tailored therapy in the future.

## NEODAJUVANT CHEMOTHERAPY *VS* CHEMORADIATION

A thorough review of the literature supports the use of neoadjuvant chemotherapy and radiation (nCRT) followed by surgery for EAC over surgery alone [8, 9], or even neoadjuvant chemotherapy (nCTX) alone followed by surgery with a reported 25-30% pCR rate in nCRT trials and < 10% in the nCTX only trials [8, 10–12]. The absolute risk reduction and number needed to treat are also much higher and lower, respectively, in the nCRT group compared to nCTX alone group [13]. The fact that nCRT has a better response rate than nCTX alone suggests that there must be a synergistic effect between chemotherapy and radiation such that concurrent therapy results in the best outcome [14, 15]. It is well known that patients with pCR when compared to non-pCR after neoadjuvant treatment have a higher rate of R0 resection and lower rate of tumor recurrence, as well as improved disease-free interval and overall survival compared to the non-pCR group [7, 16]. This use of dual modality is intuitively an appealing strategy since it allows treatment of micrometastatic disease while tumor blood supply is still intact when chemotherapy can arrest the tumor cells in a certain growth phase such that the radiation can exert it's toxic effects [17, 18].

**Figure 2:**
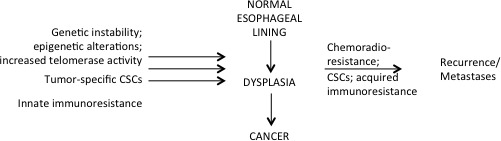
The complexity of tumorigenesis While multiple hypotheses exists regarding what results in cancer development, equally large number of hypotheses exist to explain tumor recurrences and development of distant metastases as well as chemoradio-resistance. For instance, cancer stem cells are attributed to give rise to cancer, but they have also been shown to play a role in chemoradio-resistance and hence tumor recurrence. Similarly, several different genetic and epigenetic silencing pathways have been linked to carcinogenesis, while other epigenetic silencing pathways have been associated with enhanced cell survival, ongoing tumor growth, and metastases with simultaneous escape mechanisms acquired against chemoradiation and immunity. Abbreviations: CSCs, cancer stem cells.

The role of cancer stem cells, genetics, epigenetics, and immunotherapy in EAC, as well as the identification of potential biomarkers to predict tumor progression and treatment response are in various exploratory phases (Figure 2). For the rest of this paper, we will first discuss the current treatment regimens available for EAC and then focus on unchartered grounds and future direction of cancer research that may make an impact in the treatment of patients with EAC and overall pCR rate.

## CURRENT CHEMORADIATION AND BIOLOGICAL THERAPY REGIMENS

Currently, neoadjuvant chemotherapy regimens available for the treatment of EAC include 5-fluorouracil, platinum agent, irinotecan, and taxanes. Typical preoperative radiation dose ranges from 44 to 50.4 Gy as radiation over 50.4 Gy does not impact tumor response and indeed only results in more normal tissue toxicity (INT 0123 trial) [19]. The mechanism behind this concurrent therapy is that the chemotherapy sensitizes the tumor to radiation and arrests the cells by synchronizing them to G0/G1 phase where radiation can be most effective [18].

Besides these cytotoxic chemotherapy agents, there are very limited biological agents currently approved for the treatment of esophageal cancer and the only two currently FDA-approved (trastuzumab and ramucirumab) are for patients with locally advanced unresectable or metastatic disease. Trastuzumab (Herceptin), a monoclonal antibody against human epidermal growth factor receptor type 2 (Her-2), was studied in a multi-institutional ToGA trial where almost 600 patients were enrolled in a randomized phase III trial [20]. All patients enrolled had Her-2/neu overexpression and the results demonstrated a significant overall survival benefit over standard chemotherapy (13.8 *vs* 11.1 months, *p* = 0.0046). Similarly, ramucirumab, a monoclonal antibody against human vascular endothelial growth factor receptor 2 (VEGFR2), was approved for the treatment of advanced gastric or GEJ adenocarcinoma after disease progression on first-line therapy. Two large randomized international multicenter phase III trials (REGARD and RAINBOW) demonstrated some survival benefit with ramucirumab (5.2 *vs* 3.8 months, *p* = 0.047 and 9.6 *vs* 7.4 months, *p* = 0.017) [21, 22]. Despite these large randomized trials, the overall benefits of these two biological agents are marginally significant with survival difference of merely 2-3 months.

Unlike Her2 and VEGF, other biological agents targeting epidermal growth factor receptor (EGFR), fibroblast growth factor receptor (FGFR), insulin growth factor receptor (IGF-R), c-Met, PI3K, as well as poly [adenosine diphosphate (ADP)]-ribose polymerase (PARP) inhibitors have been explored in pre-clinical settings and have been under investigation in various Phase I-III clinical trials [23, 24]. However, none of these have been able to demonstrate significant improvements in efficacy and have therefore not been approved by the FDA.

While looking for candidate genes is the most common method of exploring biological agents, another approach to analyze the biology of tumors would be to study them backwards. In other words, it would be prudent to study the altered molecular pathways of the complete responders to see what biological theme they have in common or even study the long-term (5-year) survivors *versus* the short-term survivors to explain tumor biology. Given the significant rise in incidence of EAC with simultaneous limitation of robust biological agents, the need for additional targeted agents is timely warranted. However, the heterogeneity of EAC lends itself to variable tumor response in different patients, which then compels clinicians to molecularly profile each patient and perhaps identify tumor biomarkers that can predict which patient will benefit from which targeted therapy and follow tumor response.

## CANCER STEM CELLS

Cancer stem cells (CSCs) are believed to be that sub-population of cells found in tumors that retain the capacity to self-renew and differentiate into a heterogeneous population of cells in an anchorage-independent manner [25, 26]. They have been defined differently by different authors, either by cell surface marker such as CD133, side population, or ALDH-1 (aldehyde dehydrogenase-1) based assay [27–29]. Many of the studies on the existence of cancer stem cells (CSCs) have been done on glioma, breast cancer, colon cancer, carcinoid tumors, and melanoma.

Although a paradigm shift in understanding carcinogenesis, limited studies have been performed on CSCs in EAC to date. In 2014, Ajani et al demonstrated how ALDH-1 expression correlated with presence of CSCs in EAC patients and also predicted tumor response to nCRT [30]. Those patients with pCR had minimal expression of ALDH-1, however patients extremely resistant to nCRT had very high levels of ALDH-1, which correlated with a higher incidence of death or relapse (HR = 3.87;*p* = 0.006) and nodal metastases. The results of this study implicated CSCs to play a role in tumor chemoradio-resistance and response to CRT. Using three EAC cell lines, the study also demonstrated a linear association between ALDH-1 levels and tumorigenicity, thus suggesting the use of ALDH-1 as a biomarker to predict tumor response [30]. Furthermore, Honjo et al identified these ALDH-1 positive cells in both EAC and ESCC cell lines and demonstrated how metformin (an inhibitor of the mammalian target of rapamycin pathway) has an increased activity against esophageal cancer because it is able to specifically target the CSCs [31]. In this study, metformin was able to inhibit tumor cell growth, induce apoptosis, and sensitize the cells to chemotherapy agents like 5-fluorouracil. Additionally, metformin successfully diminished the ability of EAC cell lines to develop tumor spheres as well as reduced the fraction of ALDH-1 positive cells, suggesting that it can be used to target the CSCs.

It is understood and validated that CSCs play a role in tumor progression, metastasis, and resistance to chemoradiation. Indeed, CSCs have been shown to have increased expression of adhesion and drug-efflux genes such that they have higher potential for epithelial-mesenchymal transition and develop chemoresistance in comparison to its counterparts [32]. Radio-resistant EAC and ESCC CSCs also have increased telomerase activity when compared to radio-naïve cells (data extrapolated from cell lines), and therefore possess a distinct survival advantage [33]. This is consistent with the dogma that telomerase activity can be a measure of radiocurability in tumor cells [34]. Zhang et al further demonstrated how a telomerase-specific oncolytic adenovirus was able to target radioresistant CSC-like cells since they were telomerase rich while sparing the normal cells, thus suggesting that human TERT (telomerase reverse transcriptase, catalytic unit of telomerase, the enzyme that maintains telomeric DNA) can be used for targeted therapy of these CSCs [33, 35]. Consistent with these results are that DNA damage response proteins, like single-strand DNA-binding protein SSB1, which plays a critical role in the recruitment of hTERT to the telomere G-overhang [36], may offer additional therapeutic targets in EAC. Stem cells have been identified in ESCC lines as well, where a subpopulation of cells termed as side-population had the capacity to self-renew and express elevated levels of hTERT (with higher telomerase activity), Oct-4, SOX-2, BMI, ZFX, ABC transporters, and multiple anti-apoptotic and pro-survival Wnt and Notch signal pathway-related genes [29]. Che et al elegantly developed an ESCC radioresistant cell line by irradiating tumor cells *in vitro* [37]. They then demonstrated how fractionated irradiation indeed enriched or selected for that fraction of cells enriched in CSC markers, thus suggesting that CSCs have enhanced resistance to radiation. Similar research has been done in glioma, where CSCs (CD133-positive cells) were able to repair DNA damage more efficiently and rapidly than CD133 negative cells [38]. Most of this cancer stem cell research has been performed in other solid organ tumors and ESCC [29, 39], with limited studies on EAC [30, 40, 41]. Recognizing that EAC tumors can harbor a CSC population that manages to escape chemotherapy and/or radiation should open opportunities for scientists to explore and understand the pivotal role of CSCs and indeed exploit the underlying mechanisms in its treatment.

## GENETICS AND EPIGENETICS

EAC tends to display significant tumor heterogeneity where different clonally-derived tumor regions can express different genetic profiles and undergo transformation from Barrett's esophagus into invasive cancer [42]. This indeed can explain why some EACs are so aggressive and difficult to treat with chemotherapy, radiation, and biological agents while others are not (referring to those 30% of patients that have a complete response). Understanding the pathways that lead to such heterogeneity is urgent. But perhaps it's not solely genetic factors that make esophageal cancer so aggressive, and indeed it may be the environment or post-translational modifications of expressed proteins that leads to differential protein expression or function. Mechanisms that contribute to EAC tumor progression and chemo-radioresistance development, other than mere genomic mutations, such as differences in epigenetics, remain under-explored.

### Role of genetics

There have been several whole genome and whole exome sequence studies in both EAC and ESCC [43, 44]. However, very few genes with mutations have been identified that can be implicated as having a role in esophageal tumor growth, and could therefore serve as potential therapeutic targets, such as tumor suppressors p27, p53, and CDKN2A [43, 45, 46]. While p53 has been the most extensively studied molecule in EAC in terms of tumor suppressor function, potential biomarker with loss of heterozygosity, and associated with neoplastic progression [47, 48], no good agent has been developed to stabilize the protein and prevent its inactivation, degradation, or epigenetic silencing. Additionally, ARID1A, PIK3CA, SMAD4, ELMO1, and DOCK2 have been identified as potentially mutated in EAC [43]; and RB1, NOTCH1, ADAM29, and FAM135B in ESCC [49]. Although gene specific research has confirmed a correlation between their mutant status and tumor growth or migration, none of these genes have become potential biological targets. Dulak et al demonstrated 4 oncogenes (CCNE1, CCND1, CDK6, and MYC) and 7 members of the tyrosine kinase/MAPK signaling pathways (EGFR, KRAS, MET, ERBB2, FGFR1, FGFR2, and IGF1R) to be aberrantly expressed in EAC tumor specimens using GeneChip Human Mapping arrays and Genome-Wide Human SNP genomic profiling arrays [50]. However, as stated above, most of these targets have been studied without meaningful results.

Telomere shortening, gene copy number alterations, and genomic rearrangements involving chromothripsis and breakage-fusion-bridge have also been implicated in EAC tumorigenesis [51–54]. Telomerase activation is associated with significant number of cancer cell types, including EAC, lung cancer, and ovarian cancer where addition of telomeric-end DNA enhances multiplication of cancer cells and protects the chromosomes from degradation [55]. Qi and Mi recently demonstrated how full-length hTERT antisense complimentary DNA when introduced into an ovarian cancer cell line effectively inhibited the corresponding growth of xenografts by arresting the cells in G0/G1 phase and thus preventing them from undergoing replication as well as decreasing telomerase activity [56]. Agarwal et al have similarly demonstrated how human telomerase RNA-targeted antisense agents also inhibit telomerase activity, thereby enhancing hyperthermia-mediated ionizing radiation induced cell killing and indeed radiosensitizing tumors [57]. Telomere uncapping, as opposed to TERT inhibition has also been demonstrated as a different mechanism to target lung cancer cells by hindering the cell's ability to interact with binding proteins and thereby resulting in cellular apoptosis [58]. Unfortunately, therapy targeting telomere maintenance can affect normal cells in principle. Treatments like G-quadruplex ligands which inhibit telomerase activity and shorten telomere length in cancer also have unexpected toxicity in normal tissue [59, 60]. However, due to the disproportional ratios of telomerase present in normal *versus* tumor cells, telomerase-targeted therapy can be an effective anticancer therapy while sparing normal tissue [59, 61]. Recently, inhibition of telomerase, and therefore shortening of telomeres, has been considered as a mechanism to target tumor growth in Barrett's induced EAC [62, 63].

While multiple mechanisms have been proposed to contribute to EAC genomic instability, and therefore oncogenesis and malignant transformation, tumor heterogeneity makes it difficult to develop a targeted therapy standard to all EAC patients, as the data cannot be generalized to all patients [54].

### Role of epigenetics

Epigenetics is a process where modification of expressed proteins, such as histones and transcriptional factors or methylation of DNA, alters the phenotypic outcome. It is believed that epigenetics plays a larger role in cancer than originally appreciated [64–66]. This indeed has opened many more avenues for researchers to explore. Whereas genetic mutations are fixed, epigenetic abnormalities can potentially be modified and corrected without targeting the genome itself. They can affect many of the molecular mechanisms involved in tumor growth such as cell cycle, DNA repair, survival and apoptosis, tumor suppressors, and epithelial to mesenchymal transformation thus affecting cell adhesion and invasion. Pathways involving epigenetics in esophageal cancer (DNA methylation, deacetylation, histone modification, and microRNA-mediated silencing of RNA), transdifferentiation, and immune resistance as well chemotherapy and XRT resistance are slowly emerging as potential targets in the future [67].

The best-studied epigenetic mechanism in EAC is DNA methylation. Methylation of 5′CpG islands in gene promoters results in transcription gene silencing [68]. Methylation of certain tumor suppressor genes has been implicated in some solid malignancies including breast and colorectal cancer [69, 70]. DNA hypermethylation of certain candidate genes, such as APC (a well characterized tumor suppressor gene), TIMP3 (gene that plays a role in tumor metastasis and invasion), TERT (associated with immortalization), SOCS-3, SOCS-1 (suppressors of cytokine signaling), and p16 (also known as CDK2NA) that play a role in tumor inhibition, occurs frequently in Barrett's esophagus and EAC [71–73]. Desmocollins play a role in cell-cell adhesion, especially in epithelial cells. Normally, they maintain cell integrity and impaired desmosomal function has been implicated in multiple diseases and carcinogenesis [74, 75]. Wang et al demonstrated that hypermethylation of the desmocollin-3 (DSC3) gene in human tissue samples and EAC cell lines correlated with advanced tumor stages and lymph node positivity and that treatment with DNA methylation inhibitors successfully restored DSC3 mRNA levels [76]. Genome-wide hypermethylation and gene methylation analyses have also been proposed as candidate methods for screening, early detection, disease progression and EAC therapy response prediction, however, none of these have been rigorously validated and reached clinical trials [77].

Hamilton et al suggested that tumor suppressor gene inactivation by methylation may be correlated to tumor response to chemoradiation in esophageal cancer [78]. In their study, a methylation-specific polymerase chain reaction analysis of esophageal cancer DNA from patients treated with chemoradiation was performed and the results confirmed that promoter methylation levels in patients that responded to treatment were lower than in patient DNA from nonresponders, thus suggesting that methylation status can be used as a potential biomarker to predict tumor response to chemoradiation [78]. Kaz et al very neatly demonstrated in another more recent publication how Barrett's and EAC carry differentially methylated CpG sites and how Barrett's, dysplastic epithelium, and EAC have distinct characteristic heat maps [79]. Indeed, when these differentially methylated CpG sites were mapped to biological processes, pathways related to cell proliferation and migration were over-represented in EAC and those regulating cell cycle and immune system processes were under-represented, thus suggesting their role in tumor development [79].

Epigenetic modifiers, such as DNA cytosine-5-methyltransferase I (DNMT1) and class I histone deacetylase 1 and 2 (HDAC1/2), are associated with EAC and combined inhibition of both DNMT and HDAC has been demonstrated to be an effective strategy to induce DNA damage *in vitro* that results in cell viability loss, enhanced apoptosis, and decreased cell migration [80, 81]. It has been well established that loss of the p53 allele also correlates with development of Barrett's and its progression to low-grade dysplasia, high-grade dysplasia, and eventually invasive adenocarcinoma [45]. P53 is a tumor suppressor protein and acts as a checkpoint in cell cycle progression, thereby regulating cell quiescence, proliferation, and growth arrest. Miyashita et al demonstrated that HDAC1 deacetylates p53, which then results in inhibition of apoptosis thus allowing tumor growth [82]. Their results also suggest that HDAC inhibitors can arrest EAC tumor growth and induce apoptosis.

A role for microRNAs in Barrett's esophagus and carcinogenesis has recently been described [83, 84]. MicroRNAs are a newly discovered class of small non-coding 21-nucleotide RNA molecules that regulate gene expression by binding to their corresponding mRNA and therefore, inhibit their expression or translation. Indeed, they have been shown to control the expression of oncogenes and tumor suppressor genes in other solid malignancies including gastric cancer, hepatocellular carcinoma and breast cancer [85–87], thus making them potent regulators of tumorigenesis and potential biomarkers. The role of micro-RNAs has now also been elucidated in Barrett's esophagus and its progression into cancer [88, 89]. Up-regulated miR-21 and miR-194 levels have been correlated with intestinal differentiation of Barrett's into EAC, tumor development and progression, and development of metastases [88]. The microRNAs miR-143, miR-145, miR-203, miR-215, and miR-518b on the other hand, are down-regulated in EAC where they normally serve as tumor suppressor genes and their down-regulation results in overexpression of oncogenes [88, 89]. In 2011, Bansal et al were able to discriminate between Barrett's esophagus with dysplasia and without dysplasia with reasonable clinical accuracy using a panel of selected miRNAs, thus demonstrating the feasibility of using miRNAs as biomarkers [90]. Micro-RNAs have therefore become yet another potential field of research in developing EAC-specific targeted therapy and prognostic indicators.

## IMMUNOTHERAPY

Extensive research has been conducted on the relationship between the immune system and cancer in recent years with the goal of developing new immunotherapy-based treatments. The dramatic success of the approach was first evident in melanoma where agents were successfully developed to enhance the immune response against it [91]. The basic concept behind this mechanism is that tumors affect the patient's immune system by inhibiting T-cell functions that normally suppress and eliminate transformed cells, thereby allowing tumor growth and metastasis. Therefore, antibodies and drugs have been in development that can block the inhibition of such immune checkpoints.

Cytotoxic T-lymphocyte-associated antigen (CTLA-4) is an early immune response element, and when bound to its co-stimulatory proteins CD80 (B7-1) or CD86 (B7-2), down regulates immunity and negatively regulates T cell activation [92]. CTLA-4 inhibits CD4 helper T-cell function and stimulates CD4 T-regulatory cells [93]. Similarly, programmed cell death 1 (PD-1) is a T-cell surface receptor that inhibits T-cell function when bound to its ligands programmed cell death ligand 1 (PDL-1, B7-H1) or PD-L2 (B7-DC) [94]. PD-1 negatively regulates T-cell activation *via* a distinct mechanism from CTLA-4, although the end result is the same [94, 95]. Antibody-based agents like ipilimumab, lambrolizumab, and nivolumab are, therefore, used to block CTLA-4 and PD-1 binding to PDL-1 respectively, which ultimately enhances endogenous immune responses and antitumor activity. This concept is also being explored in other malignancies with appreciable results in non small cell lung cancer, renal cell cancer, colorectal cancer, and prostate cancer [96]. Esophageal and gastric malignancies are also being explored in terms of immune checkpoint inhibition trials and early results demonstrate penetrance of immune targets into esophageal squamous cell carcinoma (ESCC) and gastric adenocarcinoma [97]. Since irradiation is known to induce antigen expression and upregulate PD-L1 expression [98, 99], there is potential for concomitant use of immunotherapy and chemoradiation in a neoadjuvant setting in esophageal cancer. EAC in general, however, has been a challenging malignancy in terms of immune targets and little research has been published on it.

## CHEMO AND RADIORESISTANCE

As highlighted above in the various sections, cancer development, and especially progression despite appropriate systemic or local therapy, has been attributed to either innate or escape mechanisms that patients either have or develop which indeed leads to cancer. While some solid tumors have demonstrated aberrant genetic mutations, others have acquired resistance. For example, deregulated or elevated phosphatidylinositol 3-kinse (PI3K)/AKT activity has been reported in several tumors and even linked to radioresistance [100, 101]. Similarly, some evidence also suggests a role of activated PI3K and its downstream effector AKT in EAC [102, 103]. Therefore, targeting PI3K in EAC has been shown to inhibit tumor cell proliferation, enhance apoptosis, inhibit *in vivo* tumor growth, and affect DNA damage response and repair pathways [104]. Inhibition of PI3K has also been associated with enhanced sensitivity of cancer cells to ionizing radiation in other malignancies [105, 106], which yet needs to be confirmed in EAC.

Typically, ionizing radiation leads to cell death by producing double strand breaks (DSBs). A hallmark of DNA DSB recognition and repair is histone H2A phosphorylation, which is thought to mark sites of DNA damage. This lethal DNA damage is then either repaired by the error prone non-homologous end joining (NHEJ) through direct ligation of the DSB ends in the G0/G1 phase of the cell cycle, or by the error-free homologous recombination (HR) mechanism in the S and G2 phase by utilizing the sister chromatid as a template for repair, which accurately restores the genomic sequence [107].

There is a very close association between HR and telomerase in EAC. As discussed above, telomerase provides cancer cells an unlimited lifespan [35]. And although one would think that telomerase inhibition should lead to shorter telomeres and therefore increased apoptosis, research has demonstrated that telomerase inhibition leads to increased HR activity and RAD51 expression, which then serves to stabilize the genome thus attenuating the intended efficacy of telomerase inhibition [63]. Therefore, telomerase inhibition alone does not affect tumor growth in EAC unless combined with HR inhibition with knockdown or inhibition of RAD51, which then leads to enhanced radiosensitivity [63, 108].

## SUMMARY

As the genetic and molecular basis behind EAC becomes clearer with continued research, the standard treatment paradigm continues to evolve for patients with locally advanced EAC. Since routine use of neoadjuvant chemoradiation has not made a significant impact on patient outcomes, novel approaches need to be explored to sensitize these aggressive tumors to chemoradiation and improve clinical response rate. However, given the heterogenous nature and responses of tumors to various biological treatments, a patient-tailored cancer treatment is required. In the future, molecular profiling will be warranted to identify specific patients who may benefit from specific targeted therapies. The development of such personalized cancer treatments may significantly impact the pCR rate of EAC patients.

## References

[1] Prithviraj GK, Baksh K, Fulp W, Meredith K, Hoffe S, Shridhar R, Almhanna K (2014). Carboplatin and paclitaxel as first-line treatment of unresectable or metastatic esophageal or gastric cancer. Dis Esophagus.

[2] National Cancer Institute Surveillance, Epidemiology, and End Results Program.

[3] Gaur P, Kim MP, Dunkin BJ (2014). Esophageal cancer: Recent advances in screening, targeted therapy, and management. J Carcinog.

[4] Shapiro J, van Lanschot JJ, Hulshof MC, van Hagen P, van Berge Henegouwen MI, Wijnhoven BP, van Laarhoven HW, Nieuwenhuijzen GA, Hospers GA, Bonenkamp JJ, Cuesta MA, Blaisse RJ, Busch OR (2015). Neoadjuvant chemoradiotherapy plus surgery versus surgery alone for oesophageal or junctional cancer (CROSS): long-term results of a randomised controlled trial. Lancet Oncol.

[5] Courrech Staal EF, Aleman BM, Boot H, van Velthuysen ML, van Tinteren H, van Sandick JW (2010). Systematic review of the benefits and risks of neoadjuvant chemoradiation for oesophageal cancer. Br J Surg.

[6] Tepper J, Krasna MJ, Niedzwiecki D, Hollis D, Reed CE, Goldberg R, Kiel K, Willett C, Sugarbaker D, Mayer R (2008). Phase III trial of trimodality therapy with cisplatin, fluorouracil, radiotherapy, and surgery compared with surgery alone for esophageal cancer: CALGB 9781. J Clin Oncol.

[7] Meredith KL, Weber JM, Turaga KK, Siegel EM, McLoughlin J, Hoffe S, Marcovalerio M, Shah N, Kelley S, Karl R (2010). Pathologic response after neoadjuvant therapy is the major determinant of survival in patients with esophageal cancer. Ann Surg Oncol.

[8] van Hagen P, Hulshof MC, van Lanschot JJ, Steyerberg EW, van Berge Henegouwen MI, Wijnhoven BP, Richel DJ, Nieuwenhuijzen GA, Hospers GA, Bonenkamp JJ, Cuesta MA, Blaisse RJ, Busch OR (2012). Preoperative chemoradiotherapy for esophageal or junctional cancer. N Engl J Med.

[9] Walsh TN, Noonan N, Hollywood D, Kelly A, Keeling N, Hennessy TP (1996). A comparison of multimodal therapy and surgery for esophageal adenocarcinoma. N Engl J Med.

[10] IBurmeister BH, Thomas JM, Burmeister EA, Walpole ET, Harvey JA, Thomson DB, Barbour AP, Gotley DC, Smithers BM (2011). Is concurrent radiation therapy required in patients receiving preoperative chemotherapy for adenocarcinoma of the oesophagus? A randomised phase II trial. Eur J Cancer.

[11] Sjoquist KM, Burmeister BH, Smithers BM, Zalcberg JR, Simes RJ, Barbour A, Gebski V, Australasian Gastro-Intestinal Trials G (2011). Survival after neoadjuvant chemotherapy or chemoradiotherapy for resectable oesophageal carcinoma: an updated meta-analysis. Lancet Oncol.

[12] Stahl M, Walz MK, Stuschke M, Lehmann N, Meyer HJ, Riera-Knorrenschild J, Langer P, Engenhart-Cabillic R, Bitzer M, Konigsrainer A, Budach W, Wilke H (2009). Phase III comparison of preoperative chemotherapy compared with chemoradiotherapy in patients with locally advanced adenocarcinoma of the esophagogastric junction. J Clin Oncol.

[13] Gebski V, Burmeister B, Smithers BM, Foo K, Zalcberg J, Simes J, Australasian Gastro-Intestinal Trials G (2007). Survival benefits from neoadjuvant chemoradiotherapy or chemotherapy in oesophageal carcinoma: a meta-analysis. Lancet Oncol.

[14] Denham JW, Burmeister BH, Lamb DS, Spry NA, Joseph DJ, Hamilton CS, Yeoh E, O'Brien P, Walker QJ (1996). Factors influencing outcome following radio-chemotherapy for oesophageal cancer. The Trans Tasman Radiation Oncology Group (TROG). Radiother Oncol.

[15] Urschel JD, Vasan H (2003). A meta-analysis of randomized controlled trials that compared neoadjuvant chemoradiation and surgery to surgery alone for resectable esophageal cancer. Am J Surg.

[16] Chirieac LR, Swisher SG, Ajani JA, Komaki RR, Correa AM, Morris JS, Roth JA, Rashid A, Hamilton SR, Wu TT (2005). Posttherapy pathologic stage predicts survival in patients with esophageal carcinoma receiving preoperative chemoradiation. Cancer.

[17] Hennequin C, Favaudon V (2002). Biological basis for chemo-radiotherapy interactions. Eur J Cancer.

[18] Kleinberg L, Gibson MK, Forastiere AA (2007). Chemoradiotherapy for localized esophageal cancer: regimen selection and molecular mechanisms of radiosensitization. Nat Clin Pract Oncol.

[19] Minsky BD, Pajak TF, Ginsberg RJ, Pisansky TM, Martenson J, Komaki R, Okawara G, Rosenthal SA, Kelsen DP (2002). INT 0123 (Radiation Therapy Oncology Group 94-05) phase III trial of combined-modality therapy for esophageal cancer: high-dose versus standard-dose radiation therapy. J Clin Oncol.

[20] Bang YJ, Van Cutsem E, Feyereislova A, Chung HC, Shen L, Sawaki A, Lordick F, Ohtsu A, Omuro Y, Satoh T, Aprile G, Kulikov E, Hill J (2010). Trastuzumab in combination with chemotherapy versus chemotherapy alone for treatment of HER2-positive advanced gastric or gastro-oesophageal junction cancer (ToGA): a phase 3, open-label, randomised controlled trial. Lancet.

[21] Fuchs CS, Tomasek J, Yong CJ, Dumitru F, Passalacqua R, Goswami C, Safran H, dos Santos LV, Aprile G, Ferry DR, Melichar B, Tehfe M, Topuzov E (2014). Ramucirumab monotherapy for previously treated advanced gastric or gastro-oesophageal junction adenocarcinoma (REGARD): an international, randomised, multicentre, placebo-controlled, phase 3 trial. Lancet.

[22] Wilke H, Muro K, Van Cutsem E, Oh SC, Bodoky G, Shimada Y, Hironaka S, Sugimoto N, Lipatov O, Kim TY, Cunningham D, Rougier P, Komatsu Y (2014). Ramucirumab plus paclitaxel versus placebo plus paclitaxel in patients with previously treated advanced gastric or gastro-oesophageal junction adenocarcinoma (RAINBOW): a double-blind, randomised phase 3 trial. Lancet Oncol.

[23] Kothari N, Almhanna K (2015). Current status of novel agents in advanced gastroesophageal adenocarcinoma. J Gastrointest Oncol.

[24] Crosby T, Hurt CN, Falk S, Gollins S, Mukherjee S, Staffurth J, Ray R, Bashir N, Bridgewater JA, Geh JI, Cunningham D, Blazeby J, Roy R (2013). Chemoradiotherapy with or without cetuximab in patients with oesophageal cancer (SCOPE1): a multicentre, phase 2/3 randomised trial. Lancet Oncol.

[25] Jordan CT, Guzman ML, Noble M (2006). Cancer stem cells. N Engl J Med.

[26] Ricci-Vitiani L, Lombardi DG, Pilozzi E, Biffoni M, Todaro M, Peschle C, De Maria R (2007). Identification and expansion of human colon-cancer-initiating cells. Nature.

[27] Gaur P, Sceusi EL, Samuel S, Xia L, Fan F, Zhou Y, Lu J, Tozzi F, Lopez-Berestein G, Vivas-Mejia P, Rashid A, Fleming JB, Abdalla EK (2011). Identification of cancer stem cells in human gastrointestinal carcinoid and neuroendocrine tumors. Gastroenterology.

[28] Nguyen GH, Murph MM, Chang JY (2011). Cancer stem cell radioresistance and enrichment: where frontline radiation therapy may fail in lung and esophageal cancers. Cancers (Basel).

[29] Huang D, Gao Q, Guo L, Zhang C, Jiang W, Li H, Wang J, Han X, Shi Y, Lu SH (2009). Isolation and identification of cancer stem-like cells in esophageal carcinoma cell lines. Stem Cells Dev.

[30] Ajani JA, Wang X, Song S, Suzuki A, Taketa T, Sudo K, Wadhwa R, Hofstetter WL, Komaki R, Maru DM, Lee JH, Bhutani MS, Weston B (2014). ALDH-1 expression levels predict response or resistance to preoperative chemoradiation in resectable esophageal cancer patients. Mol Oncol.

[31] Honjo S, Ajani JA, Scott AW, Chen Q, Skinner HD, Stroehlein J, Johnson RL, Song S (2014). Metformin sensitizes chemotherapy by targeting cancer stem cells and the mTOR pathway in esophageal cancer. Int J Oncol.

[32] Zhang Y, Zhang X, Wang J, Shen Y, Tang X, Yu F, Wang R (2015). Expression and Function of PPARs in Cancer Stem Cells. Curr Stem Cell Res Ther.

[33] Zhang X, Komaki R, Wang L, Fang B, Chang JY (2008). Treatment of radioresistant stem-like esophageal cancer cells by an apoptotic gene-armed, telomerase-specific oncolytic adenovirus. Clin Cancer Res.

[34] Sawant SG, Gregoire V, Dhar S, Umbricht CB, Cvilic S, Sukumar S, Pandita TK (1999). Telomerase activity as a measure for monitoring radiocurability of tumor cells. FASEB J.

[35] Blackburn EH, Greider CW, Henderson E, Lee MS, Shampay J, Shippen-Lentz D (1989). Recognition and elongation of telomeres by telomerase. Genome.

[36] Pandita RK, Chow TT, Udayakumar D, Bain AL, Cubeddu L, Hunt CR, Shi W, Horikoshi N, Zhao Y, Wright WE, Khanna KK, Shay JW, Pandita TK (2015). Single-strand DNA-binding protein SSB1 facilitates TERT recruitment to telomeres and maintains telomere G-overhangs. Cancer Res.

[37] Che SM, Zhang XZ, Liu XL, Chen X, Hou L (2011). The radiosensitization effect of NS398 on esophageal cancer stem cell-like radioresistant cells. Dis Esophagus.

[38] Bao S, Wu Q, McLendon RE, Hao Y, Shi Q, Hjelmeland AB, Dewhirst MW, Bigner DD, Rich JN (2006). Glioma stem cells promote radioresistance by preferential activation of the DNA damage response. Nature.

[39] Almanaa TN, Geusz ME, Jamasbi RJ (2013). A new method for identifying stem-like cells in esophageal cancer cell lines. J Cancer.

[40] Taylor C, Loomans HA, Le Bras GF, Koumangoye RB, Romero-Morales AI, Quast LL, Zaika AI, El-Rifai W, Andl T, Andl CD (2015). Activin a signaling regulates cell invasion and proliferation in esophageal adenocarcinoma. Oncotarget.

[41] Wang Z, Da Silva TG, Jin K, Han X, Ranganathan P, Zhu X, Sanchez-Mejias A, Bai F, Li B, Fei DL, Weaver K, Carpio RV, Moscowitz AE (2014). Notch signaling drives stemness and tumorigenicity of esophageal adenocarcinoma. Cancer Res.

[42] Ross-Innes CS, Becq J, Warren A, Cheetham RK, Northen H, O'Donovan M, Malhotra S, di Pietro M, Ivakhno S, He M, Weaver JM, Lynch AG, Kingsbury Z (2015). Whole-genome sequencing provides new insights into the clonal architecture of Barrett's esophagus and esophageal adenocarcinoma. Nat Genet.

[43] Dulak AM, Stojanov P, Peng S, Lawrence MS, Fox C, Stewart C, Bandla S, Imamura Y, Schumacher SE, Shefler E, McKenna A, Carter SL, Cibulskis K (2013). Exome and whole-genome sequencing of esophageal adenocarcinoma identifies recurrent driver events and mutational complexity. Nat Genet.

[44] Weaver JM, Ross-Innes CS, Shannon N, Lynch AG, Forshew T, Barbera M, Murtaza M, Ong CA, Lao-Sirieix P, Dunning MJ, Smith L, Smith ML, Anderson CL (2014). Ordering of mutations in preinvasive disease stages of esophageal carcinogenesis. Nat Genet.

[45] Kastelein F, Biermann K, Steyerberg EW, Verheij J, Kalisvaart M, Looijenga LH, Stoop HA, Walter L, Kuipers EJ, Spaander MC, Bruno MJ, ProBar-study g (2013). Aberrant p53 protein expression is associated with an increased risk of neoplastic progression in patients with Barrett's oesophagus. Gut.

[46] Singh SP, Lipman J, Goldman H, Ellis FH, Aizenman L, Cangi MG, Signoretti S, Chiaur DS, Pagano M, Loda M (1998). Loss or altered subcellular localization of p27 in Barrett's associated adenocarcinoma. Cancer Res.

[47] Sikkema M, Kerkhof M, Steyerberg EW, Kusters JG, van Strien PM, Looman CW, van Dekken H, Siersema PD, Kuipers EJ (2009). Aneuploidy and overexpression of Ki67 and p53 as markers for neoplastic progression in Barrett's esophagus: a case-control study. Am J Gastroenterol.

[48] Weston AP, Banerjee SK, Sharma P, Tran TM, Richards R, Cherian R (2001). p53 protein overexpression in low grade dysplasia (LGD) in Barrett's esophagus: immunohistochemical marker predictive of progression. Am J Gastroenterol.

[49] Song Y, Li L, Ou Y, Gao Z, Li E, Li X, Zhang W, Wang J, Xu L, Zhou Y, Ma X, Liu L, Zhao Z (2014). Identification of genomic alterations in oesophageal squamous cell cancer. Nature.

[50] Dulak AM, Schumacher SE, van Lieshout J, Imamura Y, Fox C, Shim B, Ramos AH, Saksena G, Baca SC, Baselga J, Tabernero J, Barretina J, Enzinger PC (2012). Gastrointestinal adenocarcinomas of the esophagus, stomach, and colon exhibit distinct patterns of genome instability and oncogenesis. Cancer Res.

[51] Goh XY, Rees JR, Paterson AL, Chin SF, Marioni JC, Save V, O'Donovan M, Eijk PP, Alderson D, Ylstra B, Caldas C, Fitzgerald RC (2011). Integrative analysis of array-comparative genomic hybridisation and matched gene expression profiling data reveals novel genes with prognostic significance in oesophageal adenocarcinoma. Gut.

[52] Frankel A, Armour N, Nancarrow D, Krause L, Hayward N, Lampe G, Smithers BM, Barbour A (2014). Genome-wide analysis of esophageal adenocarcinoma yields specific copy number aberrations that correlate with prognosis. Genes Chromosomes Cancer.

[53] Shiraishi H, Mikami T, Aida J, Nakamura K, Izumiyama-Shimomura N, Arai T, Watanabe M, Okayasu I, Takubo K (2009). Telomere shortening in Barrett's mucosa and esophageal adenocarcinoma and its association with loss of heterozygosity. Scand J Gastroenterol.

[54] Nones K, Waddell N, Wayte N, Patch AM, Bailey P, Newell F, Holmes O, Fink JL, Quinn MC, Tang YH, Lampe G, Quek K, Loffler KA (2014). Genomic catastrophes frequently arise in esophageal adenocarcinoma and drive tumorigenesis. Nat Commun.

[55] Meena J, Rudolph KL, Gunes C (2015). Telomere Dysfunction, Chromosomal Instability and Cancer. Recent Results Cancer Res.

[56] Qi Z, Mi R (2016). Inhibition of human telomerase reverse transcriptase in vivo and in vitro for retroviral vector-based antisense oligonucleotide therapy in ovarian cancer. Cancer Gene Ther.

[57] Agarwal M, Pandita S, Hunt CR, Gupta A, Yue X, Khan S, Pandita RK, Pratt D, Shay JW, Taylor JS, Pandita TK (2008). Inhibition of telomerase activity enhances hyperthermia-mediated radiosensitization. Cancer Res.

[58] Mancini J, Rousseau P, Castor KJ, Sleiman HF, Autexier C (2016). Platinum(II) phenanthroimidazole G-quadruplex ligand induces selective telomere shortening in A549 cancer cells. Biochimie.

[59] Crees Z, Girard J, Rios Z, Botting GM, Harrington K, Shearrow C, Wojdyla L, Stone AL, Uppada SB, Devito JT, Puri N (2014). Oligonucleotides and G-quadruplex stabilizers: targeting telomeres and telomerase in cancer therapy. Curr Pharm Des.

[60] Ruden M, Puri N (2013). Novel anticancer therapeutics targeting telomerase. Cancer Treat Rev.

[61] Mengual Gomez DL, Armando RG, Cerrudo CS, Ghiringhelli PD, Gomez DE (2016). Telomerase as a cancer target. Development of new molecules. Curr Top Med Chem.

[62] Shammas MA, Qazi A, Batchu RB, Bertheau RC, Wong JY, Rao MY, Prasad M, Chanda D, Ponnazhagan S, Anderson KC, Steffes CP, Munshi NC, De Vivo I (2008). Telomere maintenance in laser capture microdissection-purified Barrett's adenocarcinoma cells and effect of telomerase inhibition in vivo. Clin Cancer Res.

[63] Lu R, Pal J, Buon L, Nanjappa P, Shi J, Fulciniti M, Tai YT, Guo L, Yu M, Gryaznov S, Munshi NC, Shammas MA (2014). Targeting homologous recombination and telomerase in Barrett's adenocarcinoma: impact on telomere maintenance, genomic instability and tumor growth. Oncogene.

[64] Ahrens TD, Werner M, Lassmann S (2014). Epigenetics in esophageal cancers. Cell Tissue Res.

[65] Jones PA, Baylin SB (2007). The epigenomics of cancer. Cell.

[66] Kailasam A, Mittal SK, Agrawal DK (2015). Epigenetics in the Pathogenesis of Esophageal Adenocarcinoma. Clin Transl Sci.

[67] Ramzan Z, Nassri AB, Huerta S (2014). The use of imaging and biomarkers in diagnosing Barrett's esophagus and predicting the risk of neoplastic progression. Expert Rev Mol Diagn.

[68] Herman JG, Baylin SB (2003). Gene silencing in cancer in association with promoter hypermethylation. N Engl J Med.

[69] Karsli-Ceppioglu S, Dagdemir A, Judes G, Ngollo M, Penault-Llorca F, Pajon A, Bignon YJ, Bernard-Gallon D (2014). Epigenetic mechanisms of breast cancer: an update of the current knowledge. Epigenomics.

[70] Okugawa Y, Grady WM, Goel A (2015). Epigenetic Alterations in Colorectal Cancer: Emerging Biomarkers. Gastroenterology.

[71] Clement G, Braunschweig R, Pasquier N, Bosman FT, Benhattar J (2006). Methylation of APC, TIMP3, and TERT: a new predictive marker to distinguish Barrett's oesophagus patients at risk for malignant transformation. J Pathol.

[72] Tischoff I, Hengge UR, Vieth M, Ell C, Stolte M, Weber A, Schmidt WE, Tannapfel A (2007). Methylation of SOCS-3 and SOCS-1 in the carcinogenesis of Barrett's adenocarcinoma. Gut.

[73] Bian YS, Osterheld MC, Fontolliet C, Bosman FT, Benhattar J (2002). p16 inactivation by methylation of the CDKN2A promoter occurs early during neoplastic progression in Barrett's esophagus. Gastroenterology.

[74] Chen J, O'shea C, Fitzpatrick JE, Koster MI, Koch PJ (2012). Loss of Desmocollin 3 in skin tumor development and progression. Mol Carcinog.

[75] Knosel T, Chen Y, Hotovy S, Settmacher U, Altendorf-Hofmann A, Petersen I (2012). Loss of desmocollin 1-3 and homeobox genes PITX1 and CDX2 are associated with tumor progression and survival in colorectal carcinoma. Int J Colorectal Dis.

[76] Wang Q, Peng D, Zhu S, Chen Z, Hu T, Soutto M, Saad R, Zhang S, Ei-Rifai W (2014). Regulation of Desmocollin3 Expression by Promoter Hypermethylation is Associated with Advanced Esophageal Adenocarcinomas. J Cancer.

[77] Xu E, Gu J, Hawk ET, Wang KK, Lai M, Huang M, Ajani J, Wu X (2013). Genome-wide methylation analysis shows similar patterns in Barrett's esophagus and esophageal adenocarcinoma. Carcinogenesis.

[78] Hamilton JP, Sato F, Greenwald BD, Suntharalingam M, Krasna MJ, Edelman MJ, Doyle A, Berki AT, Abraham JM, Mori Y, Kan T, Mantzur C, Paun B (2006). Promoter methylation and response to chemotherapy and radiation in esophageal cancer. Clin Gastroenterol Hepatol.

[79] Kaz AM, Wong CJ, Luo Y, Virgin JB, Washington MK, Willis JE, Leidner RS, Chak A, Grady WM (2011). DNA methylation profiling in Barrett's esophagus and esophageal adenocarcinoma reveals unique methylation signatures and molecular subclasses. Epigenetics.

[80] Langer R, Mutze K, Becker K, Feith M, Ott K, Hofler H, Keller G (2010). Expression of class I histone deacetylases (HDAC1 and HDAC2) in oesophageal adenocarcinomas: an immunohistochemical study. J Clin Pathol.

[81] Ahrens TD, Timme S, Hoeppner J, Ostendorp J, Hembach S, Follo M, Hopt UT, Werner M, Busch H, Boerries M, Lassmann S (2015). Selective inhibition of esophageal cancer cells by combination of HDAC inhibitors and Azacytidine. Epigenetics.

[82] Miyashita T, Tajima H, Munemoto M, Shah FA, Harmon JW, Watanabe T, Shoji M, Okamoto K, Nakanuma S, Sakai S, Kinoshita J, Makino I, Nakamura K (2014). Impact of histone deacetylase 1 and metastasis-associated gene 1 expression in esophageal carcinogenesis. Oncol Lett.

[83] Nguyen GH, Schetter AJ, Chou DB, Bowman ED, Zhao R, Hawkes JE, Mathe EA, Kumamoto K, Zhao Y, Budhu A, Hagiwara N, Wang XW, Miyashita M (2010). Inflammatory and microRNA gene expression as prognostic classifier of Barrett's-associated esophageal adenocarcinoma. Clin Cancer Res.

[84] Wijnhoven BP, Hussey DJ, Watson DI, Tsykin A, Smith CM, Michael MZ, South Australian Oesophageal Research G (2010). MicroRNA profiling of Barrett's oesophagus and oesophageal adenocarcinoma. Br J Surg.

[85] Huan L, Bao C, Chen D, Li Y, Lian J, Ding J, Huang S, Liang L, He X (2015). MiR-127-5p targets the biliverdin reductase B/NF-kappaB pathway to suppress cell growth in hepatocellular carcinoma cells. Cancer Sci.

[86] ILi D, Li Z, Xiong J, Gong B, Zhang G, Cao C, Jie Z, Liu Y, Cao Y, Yan Y, Xiong H, Qiu L, Yang M (2015). MicroRNA-212 functions as an epigenetic-silenced tumor suppressor involving in tumor metastasis and invasion of gastric cancer through down-regulating PXN expression. Am J Cancer Res.

[87] Zheng M, Sun X, Li Y, Zuo W (2015). MicroRNA-145 inhibits growth and migration of breast cancer cells through targeting oncoprotein ROCK1. Tumour Biol.

[88] Smith CM, Watson DI, Michael MZ, Hussey DJ (2010). MicroRNAs, development of Barrett's esophagus, and progression to esophageal adenocarcinoma. World J Gastroenterol.

[89] Huang J, Zhang SY, Gao YM, Liu YF, Liu YB, Zhao ZG, Yang K (2014). MicroRNAs as oncogenes or tumour suppressors in oesophageal cancer: potential biomarkers and therapeutic targets. Cell Prolif.

[90] Bansal A, Lee IH, Hong X, Anand V, Mathur SC, Gaddam S, Rastogi A, Wani SB, Gupta N, Visvanathan M, Sharma P, Christenson LK (2011). Feasibility of mcroRNAs as biomarkers for Barrett's Esophagus progression: a pilot cross-sectional, phase 2 biomarker study. Am J Gastroenterol.

[91] Hodi FS, O'Day SJ, McDermott DF, Weber RW, Sosman JA, Haanen JB, Gonzalez R, Robert C, Schadendorf D, Hassel JC, Akerley W, van den Eertwegh AJ, Lutzky J (2010). Improved survival with ipilimumab in patients with metastatic melanoma. N Engl J Med.

[92] Linsley PS, Bradshaw J, Greene J, Peach R, Bennett KL, Mittler RS (1996). Intracellular trafficking of CTLA-4 and focal localization towards sites of TCR engagement. Immunity.

[93] Peggs KS, Quezada SA, Chambers CA, Korman AJ, Allison JP (2009). Blockade of CTLA-4 on both effector and regulatory T cell compartments contributes to the antitumor activity of anti-CTLA-4 antibodies. J Exp Med.

[94] Parry RV, Chemnitz JM, Frauwirth KA, Lanfranco AR, Braunstein I, Kobayashi SV, Linsley PS, Thompson CB, Riley JL (2005). CTLA-4 and PD-1 receptors inhibit T-cell activation by distinct mechanisms. Mol Cell Biol.

[95] Freeman GJ, Long AJ, Iwai Y, Bourque K, Chernova T, Nishimura H, Fitz LJ, Malenkovich N, Okazaki T, Byrne MC, Horton HF, Fouser L, Carter L (2000). Engagement of the PD-1 immunoinhibitory receptor by a novel B7 family member leads to negative regulation of lymphocyte activation. J Exp Med.

[96] Topalian SL, Hodi FS, Brahmer JR, Gettinger SN, Smith DC, McDermott DF, Powderly JD, Carvajal RD, Sosman JA, Atkins MB, Leming PD, Spigel DR, Antonia SJ (2012). Safety, activity, and immune correlates of anti-PD-1 antibody in cancer. N Engl J Med.

[97] Raufi AG, Klempner SJ (2015). Immunotherapy for advanced gastric and esophageal cancer: preclinical rationale and ongoing clinical investigations. J Gastrointest Oncol.

[98] Deng L, Liang H, Burnette B, Beckett M, Darga T, Weichselbaum RR, Fu YX (2014). Irradiation and anti-PD-L1 treatment synergistically promote antitumor immunity in mice. J Clin Invest.

[99] Zhang B, Bowerman NA, Salama JK, Schmidt H, Spiotto MT, Schietinger A, Yu P, Fu YX, Weichselbaum RR, Rowley DA, Kranz DM, Schreiber H (2007). Induced sensitization of tumor stroma leads to eradication of established cancer by T cells. J Exp Med.

[100] Yap TA, Garrett MD, Walton MI, Raynaud F, de Bono JS, Workman P (2008). Targeting the PI3K-AKT-mTOR pathway: progress, pitfalls, and promises. Curr Opin Pharmacol.

[101] Zhan M, Han ZC (2004). Phosphatidylinositide 3-kinase/AKT in radiation responses. Histol Histopathol.

[102] Phillips WA, Russell SE, Ciavarella ML, Choong DY, Montgomery KG, Smith K, Pearson RB, Thomas RJ, Campbell IG (2006). Mutation analysis of PIK3CA and PIK3CB in esophageal cancer and Barrett's esophagus. Int J Cancer.

[103] Beales IL, Ogunwobi O, Cameron E, El-Amin K, Mutungi G, Wilkinson M (2007). Activation of Akt is increased in the dysplasia-carcinoma sequence in Barrett's oesophagus and contributes to increased proliferation and inhibition of apoptosis: a histopathological and functional study. BMC Cancer.

[104] Pal J, Fulciniti M, Nanjappa P, Buon L, Tai YT, Tassone P, Munshi NC, Shammas MA (2012). Targeting PI3K and RAD51 in Barrett's adenocarcinoma: impact on DNA damage checkpoints, expression profile and tumor growth. Cancer Genomics Proteomics.

[105] Schuurbiers OC, Kaanders JH, van der Heijden HF, Dekhuijzen RP, Oyen WJ, Bussink J (2009). The PI3-K/AKT-pathway and radiation resistance mechanisms in non-small cell lung cancer. J Thorac Oncol.

[106] Zhang T, Cui GB, Zhang J, Zhang F, Zhou YA, Jiang T, Li XF (2010). Inhibition of PI3 kinases enhances the sensitivity of non-small cell lung cancer cells to ionizing radiation. Oncol Rep.

[107] Ceccaldi R, Rondinelli B, D'Andrea AD (2015). Repair Pathway Choices and Consequences at the Double-Strand Break. Trends Cell Biol.

[108] Taki T, Ohnishi T, Yamamoto A, Hiraga S, Arita N, Izumoto S, Hayakawa T, Morita T (1996). Antisense inhibition of the RAD51 enhances radiosensitivity. Biochem Biophys Res Commun.

